# *Enalikter aphson* is an arthropod: a reply to Struck *et al*. (2014)

**DOI:** 10.1098/rspb.2014.2663

**Published:** 2015-04-07

**Authors:** Derek J. Siveter, Derek E. G. Briggs, David J. Siveter, Mark D. Sutton, David Legg, Sarah Joomun

Struck *et al*. [1] raise some interesting points in contending that *Enalikter aphson* is more likely to be an annelid than an arthropod. We considered this possibility [2] but its arthropod features convinced us otherwise. Extensive use of stereo-pairs, video-slice images and virtual models [3,4] were central to our assessment [5], as for all Herefordshire Lagerstätte fossils. In preparing this reply, we have re-visited the data and we present (in the electronic supplementary material) further images in support of an arthropod assignment for *Enalikter* (electronic supplementary material, figure S1). We also respond (in the electronic supplementary material) to the comments relating to our phylogenetic analysis, which recovered a megacheiran affinity for this species.

Struck *et al*. [1] comment that the segments of *Enalikter* lack a well-delineated tergite on the dorsal side, and that the dome-like feature on the first trunk segment is not visible in lateral aspect and its structure is unclear. The dome-like tergites are not as discretely preserved as the sternites but they nonetheless appear present (electronic supplementary material, figure S1*h,l*,*m*). But even an absence of tergites would not preclude an arthropod affinity: external expression of segmentation is absent in many arthropods, e.g. in the opisthosoma of most spiders [6], or in the thorax of some cladocerans (e.g. *Bythotrephes longimanus*; see [7]). As for the structures interpreted by us as sternites, discrete ventral units are clearly present in the appropriate position corresponding to each paired trunk appendage (electronic supplementary material, figure S1*i*,*j*,*n*).

The absence of tergopleurae noted by Struck *et al*. [1] was also noted by us [5]; these structures are also lacking in some other arthropods, including *Bundenbachiellus giganteus*, which all agree is an arthropod. Tergopleurae are very susceptible to homoplastic reduction (as well as acquisition), as shown by the dataset of one of us (D.L.), which despite continuous amendment in successive papers (e.g. [5,8–10]), has consistently yielded a result indicating this. The reduction of tergopleurae is well-shown in chelicerates, particularly arachnids [11], and also in marrellomorphs [12]. Tergopleurae appear to have been acquired in the arthropod stem, in a position only slightly more basal than that recovered for megacheirans [8, fig. 3].

Struck *et al*. note that although the trunk of *Enalikter* is extremely flexible, its degree of bending is nevertheless compatible with that in other arthropods, such as centipedes. The ability to turn through a tight 180° U-bend is common to both *Enalikter* and centipedes, although it is accomplished over a greater number of body segments in centipedes, at least in those for which we have images (e.g. *Orya almohadensis*; images provided by Dr Greg Edgecombe, Natural History Museum, London). However, the contention of Struck *et al*. [1] that the *Enalikter* trunk is more reminiscent of the continuous flexibility of the skin-muscle tube in annelids is debatable. We agree that the curvature in OUMNH C.29632 appears relatively smooth and continuous, but we interpret this as a product of flexible inter-segmental regions (electronic supplementary material, figure S1*i–k*). The cuticle in these regions is concertina-like and assumes a wedge-shape (narrow on the inner side, wide on the outer) separating successive divisions of the trunk where it curves through 180°.

We maintain, in contrast to Struck *et al*. [1], that a discrete, dorsal, cap-like cover is present on the head of *Enalikter*, which represents an arthropod head shield. Its presence is evidenced where the anterior appendages clearly project from beneath the sharp margin of a dorsoventrally shallow shield (see electronic supplementary material, figure S1*a*–*c*, and also compare electronic supplementary material figure S1*c* with the analogous configuration of head shield and appendages in, for example, the isopod *Onisocryptus ovalis* (Shiino, 1942) in [13, fig. 15E]).

The clear difference between the morphology of the anterior appendages and those of the trunk of *Enalikter*, which Struck *et al*. [1] contend supports an annelid affinity, could equally well be used to support an arthropod affinity. Distinct head appendages are present in all arthropod crown groups, where this is often a defining feature. Nevertheless, in many stem-arthropods, and very rarely in crown group forms—e.g. the cephalocarid crustaceans, the posterior cephalic appendages remain similar to those of the trunk, in contrast to the more radically different prostomial appendages of annelids. Head appendages two and three of *Enalikter* are similar in morphology to the trunk appendages, differing only in size, in the development of gnathobasic endites, and reduction of the exopods. They are thus more arthropod-like than annelid-like.

The Struck *et al*. [1] assertion that an unpaired frontal appendage like that in *Enalikter* is otherwise unknown in arthropods is not strictly correct. We noted [5] the presence of a long spine in the hypostomal region of the metanauplius of the Cambrian eucrustacean *Wujicaris muelleri* that, likewise, has been compared [14] with the long pre-oral spinal process of the extant ectoparasitic fish lice *Argulus* and *Dipteropeltis*. There are some differences in detail. The spine in *Enalikter* (an adult specimen) is about 1.25 times the length of its head shield, whereas in *Wujicaris* (a juvenile specimen) it is about equal in length to its naupliar shield. Furthermore, the spine in *Wujicaris* was apparently rigid, whereas the curved whip-like structure in *Enalikter* might be considered flexible. However, if it was flexible, it should be preserved in a different disposition in different individuals, but its forwardly projecting and recurved form is the same in all three specimens. Regarding the point of attachment of this unpaired frontal feature, it appears to be attached ventrally in *Enalikter*, in contrast to a frontal or posterior position [1] in annelids.

Articulations between podomeres are not always clearly evident in Herefordshire arthropods; this is a taphonomic feature of this Lagerstätte and, to a lesser extent, an artefact resulting from the digital procedure used to retrieve the virtual models. Nevertheless, there is strong evidence for articulations where the endopods are flexed (electronic supplementary material, figure S1*f*,*g*), and the morphologically similar *Bundenbachiellus* clearly preserves jointed appendages. Although we [5] acknowledged the possibility that the filaments of the trunk appendages are controlled by turgor pressure, this does not preclude an arthropod affinity (particularly of a stem group form). The Cambrian arthropod *Marrella* appears to have filaments of similar morphology and flexibility, which may also have operated under turgor pressure [15]. Multiple filaments are well known in association with the biramous trunk limbs of arthropods (e.g. in *Naraoia longicaudata* [16, fig. 16.40c]). Each individual ray in *Enalikter* arises sequentially along the length of the outer branch, as in an arthropod exopod, rather than fanning out from the base as in annelids.

The absence of annelid-like chaetae in *Enalikter* is unlikely to reflect taphonomy or shortcomings of the reconstruction. Chaetae have a much higher preservational potential than other features of annelids [17]. Furthermore, specimens of the Herefordshire polychaete *Kenostrychus clementsi* clearly show chaetae [18,19]. The three specimens of *Enalikter* resolve fine detail such as the small, bifid terminations to the inner branches of the trunk limbs (endopods), whereas there is no evidence of chaetae.

Our observation [5] that the ventrally projecting, boss-like feature in the head of *Enalikter* recalls similar bulging structures interpreted as hypostomal homologues in *Agnostus pisiformis*, *Henningsmoenicaris scutula* and *Martinssonia elongata* [20] was based in part on a misreading of Waloszek & Müller's paper [20]. We considered *Enalikter* to lack a fully sclerotized hypostome, like that in these other three species, yet this feature was regarded by Waloszek & Müller as less sclerotized only in *M. elongata*. Our use of the word ‘recalls’ [5, p. 3] was deliberate; we did not mean to suggest that the feature is strictly identical in all these species.

The posterior of *Enalikter* may resemble the pygidium and two cirri of certain annelids [1], but its morphology also lies within the range in arthropods, and is similar to that of *Bundenbachiellus* [5].

Several other features of *Enalikter* support an arthropod affinity. The ventrally placed mouth and J-shaped gut in the head are arthropod features. The round-shaped mouth region (electronic supplementary material, figure S1*d*) is reminiscent of that in certain pan- and stem-arthropods. Head appendages two (especially) and three of *Enalikter* bear well-developed endites on their inner branches (endopods; electronic supplementary material, figure S1*e*). The bifid terminations of the trunk endopods of *Enalikter*, formed by the spinose distal tip of this branch and what appears to be the spinose end of a subterminal podomere (electronic supplementary material, figure S1*f*), are a characteristic feature of arthropods. We continue to find the overall similarity between *Enalikter* and *Bundenbachiellus* striking and maintain that the anterior head appendage of the latter is likely triflagellate ([5]; electronic supplementary material, figure S3).

The additional evidence of the morphology of *Enalikter* presented here, particularly the arthropod-like articulations of the limbs and trunk, together with the absence of any evidence for chaetae, supports our original interpretation of this animal as an arthropod. Future discoveries of other fossil arthropods will doubtless allow its relationship to other megacheirans to be resolved further.

## Supplementary Material

ESM text to accompany the Reply of Siveter et al. to the Critique of Struck et al.

## Supplementary Material

ESM figure 1.png
