# Supplementary material for: Enalikter aphson is an arthropod: a reply to Struck et al. (2014)
Source: Proc Biol Sci. 2015 Apr 7;282(1804):20142663. doi: 10.1098/rspb.2014.2663 (PMC4375861; doi:10.1098/rspb.2014.2663)
Supplement: ESM text to accompany the Reply of Siveter et al. to the Critique of Struck et al. [file rspb20142663supp1.docx]

ELECTRONIC SUPPLEMENTARY MATERIAL

We address here the questions raised by Struck *et* *al*. concerning our phylogenetic analysis and a megacheiran identity for *Enalikter*. The triflagellate antenna (first appendage) of *Enalikter* does differ from that of typical megacheirans [1], but nevertheless its morphology is consistent with that of leanchoiliids. Other hypotheses were tested during our phylogenetic analyses, and as well as coding the anterior appendages as triflagellate antennae, they were for example also coded as an unknown state. However, despite coding all potential scenarios for the homology of the anterior appendages of *Enalikter*, it continued to resolve amongst leanchoiliids, based primarily on its tagmosis.

The suggestion of Struck *et al*. that the result of our phylogenetic analysis might reflect unsupported assumptions of homology, especially with respect to the first appendage is unfounded in our view. The segmental affinity of the first (great) appendage has been discussed, with limited consensus, by numerous authors. Although these appendages are commonly interpreted as deutocerebral, based on similarities to chelicerate chelicerae, there is evidence to suggest otherwise. For instance, Tanaka *et* *al*. [2] favoured deutocerebral affinities for the great-appendages based on neurological data, but their discussion and figures suggest that the appendages they refer to are para-oral. Although deutocerebral appendages can originate from a para-oral position [3], they are (primarily) pre-oral in crown-group euarthropods [4], whereas tritocerebral appendages tend to be para-oral. In order for the great appendages to be deutocerebral, either the oral foramen or the deutocerebral neuromeres would have to be repositioned – and both these scenarios are unparsimonious regarding chelicerate affinities for megacheirans [5].

We do not have evidence to support the presence of stiff, elongate spine-like elements proximal to the flagellum in the great appendages of *Enalikter*, as in leanchoiliid megacheirans, but their absence would not necessarily preclude megacheiran affinities, as other more significant characters such as the number of head appendages still favour such an affinity. Equally, while the amount of flexure exhibited by the trunk of *Enalikter* is unusual, this does not in itself preclude megacheiran affinities. Sclerotised dorsal structures appear to be present in *Enalikter* (ESM 1*g*-*n*; see also our reply), in contrast to the interpretation of Struck *et* *al*., although the overlapping tergites typical of megacheirans are absent; this may be an autapomorphic feature of *Enalikter*. It is also worth noting that the position of *Enalikter* in our phylogenetic analyses is based on features that are present, rather than those that are absent. This is normal phylogenetic practice; phylogenies based on absence can lead to the exclusion of many taxa from their natural groups – e.g. pentastomids and barnacles from crown-group Crustacea.

The position of megacheirans within arthropods remains controversial. While Struck *et* *al*. place them within Euarthropoda, there is little evidence beyond similarities of the anterior appendages to indicate a close relationship between megacheirans and chelicerates. Other proposed features linking these taxa, such as the number of limb podomeres, are not exclusive to megacheirans and chelicerates and may represent a plesiomorphic state for Euarthropoda. The only quantitative phylogenetic analyses that resolve megacheirans as stem-chelicerates are rooted on in-group euarthropods, such as trilobitomorphs or marrellomorphs [6,7], a procedure that will necessarily resolve megacheirans as in-group euarthropods. In analyses that include a wider sampling of stem euarthropods [e.g. 8-12] megacheirans consistently resolve outside Euarthropoda, regardless of assumptions about the segmental affinities of the great-appendage. This was explored by Legg [10], who coded the great-appendages as homologous to the chelicerae of chelicerates yet failed to resolve the two as sister-taxa.

References

1. Siveter Derek J, Briggs Derek EG, Siveter David J, Sutton Mark D, Legg David, Joomun Sarah. 2014 A Silurian short-great-appendage arthropod. *Proc*. *R*. *Soc*. *B* **281**, 20132986. (doi:10.1098/rspb.2013.2986)

2. Tanaka G, Hou X-G, Ma X, Edgecombe GD, Strausfeld NJ. 2013 Chelicerate neural ground pattern in a Cambrian great appendage arthropod. *Nature* **502**, 364-367.

3. Mittmann B, Scholtz G. 2001 *Distal-less* expression in embryos of *Limulus polyphemus* (Chelicerata, Xiphosura) and *Lepisma saccharina* (Insecta, Zygentoma) suggests a role in the development of mechanoreceptors, chemoreceptors, and the CNS. *Dev*. *Genes* *Evol*. **211**, 232–243.

3. Strausfeld NJ. 2012 *Arthropod brains: evolution, functional elegance, and structural significance*. Cambridge, MA: Harvard University Press, 650 pp.

4. Legg DA. 2014 *Sanctacaris uncata*: the oldest chelicerate (Arthropoda). *Naturwissenschaften*. (doi: 10.1007/s00114-014-1245-4)

5. Cotton TJ, Braddy SJ. 2004. The phylogeny of arachnomorph arthropods and the origin of the Chelicerata. *Trans R Soc Edinb Earth Sci* **94**, 169 – 194.

6. Edgecombe GD, García-Bellido DC, Paterson JR. 2011. A new leanchoiliid megacheiran arthropod from the lower Cambrian emu Bay shale, South Australia. *Acta Palaeontol Pol* **56**, 385 – 400.

7. Budd G. 2002 A palaeontological solution to the arthropod head problem. *Nature* **417**, 27-275.

8. Daley AC, Budd GE, Caron J-B, Edgecombe GD, Collins D. 2009 The Burgess Shale anomalocaridid *Hurdia* and its significance for early euarthropod evolution. *Science* **323**, 1597-1600.

9. Legg DA. 2013 Multi-segmented arthropods from the middle Cambrian of British Columbia (Canada). *J Paleont.* **87**, 493-501.

10. Legg DA, Sutton MD, Edgecombe GD, Caron J-B. 2012 Cambrian bivalved arthropod reveals origin of arthrodization. *Proc*. *R*. *Soc*. *B* **279**, 4699-4704. (doi:10.1098/rspb.2012.1958)

11. Legg DA, Sutton MD, Edgecombe GD. 2013 Arthropod fossil data increase congruence of morphological and molecular phylogenies. *Nat.* *Comm*. **4**, 2485. (doi: 10:1038/ncomms348)

Figure 1. *a*-*n* *Enalikter* *aphson* Siveter *et* *al*., 2104. *a*-*c*, *e*, *l*-*n*, OUMNH C.29633. *a*-*c*, *e*, head area, left anterolateral ventral, anterolateral dorsal, right anterolateral ventral, posterior ventral stereo-pairs; *l*, whole specimen, anterior dorsal stereo-pair; *m*, *n*, trunk and telson area with appendages omitted, lateral and posterior ventral stereo-pairs. *d*. Isopod crustacean, *Onisocryptus* *ovalis* (Shiino, 1942) (from Vannier and Abe 1993, Figure 15E). *f*. OUMNH C.29831. (holotype), biramous trunk appendages, posterior ventral stereo-pair. *g*-*k*, OUMNH C.29632. *g*, partial trunk and endopods, exopods omitted; *h*, anterior trunk and head area, biramous appendages omitted; *i*, trunk and telson area, appendages omitted; *j*, *k*, details of trunk.

en, endite; fr, flexible inter-segmental region; hsm, head shield margin; jt, joint; st, sternite; te, tergite; tfe, terminally forked endopod. All scale bars = 1 mm.
