# Supplementary figures and images for: Enalikter aphson is an arthropod: a reply to Struck et al. (2014)
Source: Proc Biol Sci. 2015 Apr 7;282(1804):20142663. doi: 10.1098/rspb.2014.2663 (PMC4375861; doi:10.1098/rspb.2014.2663)

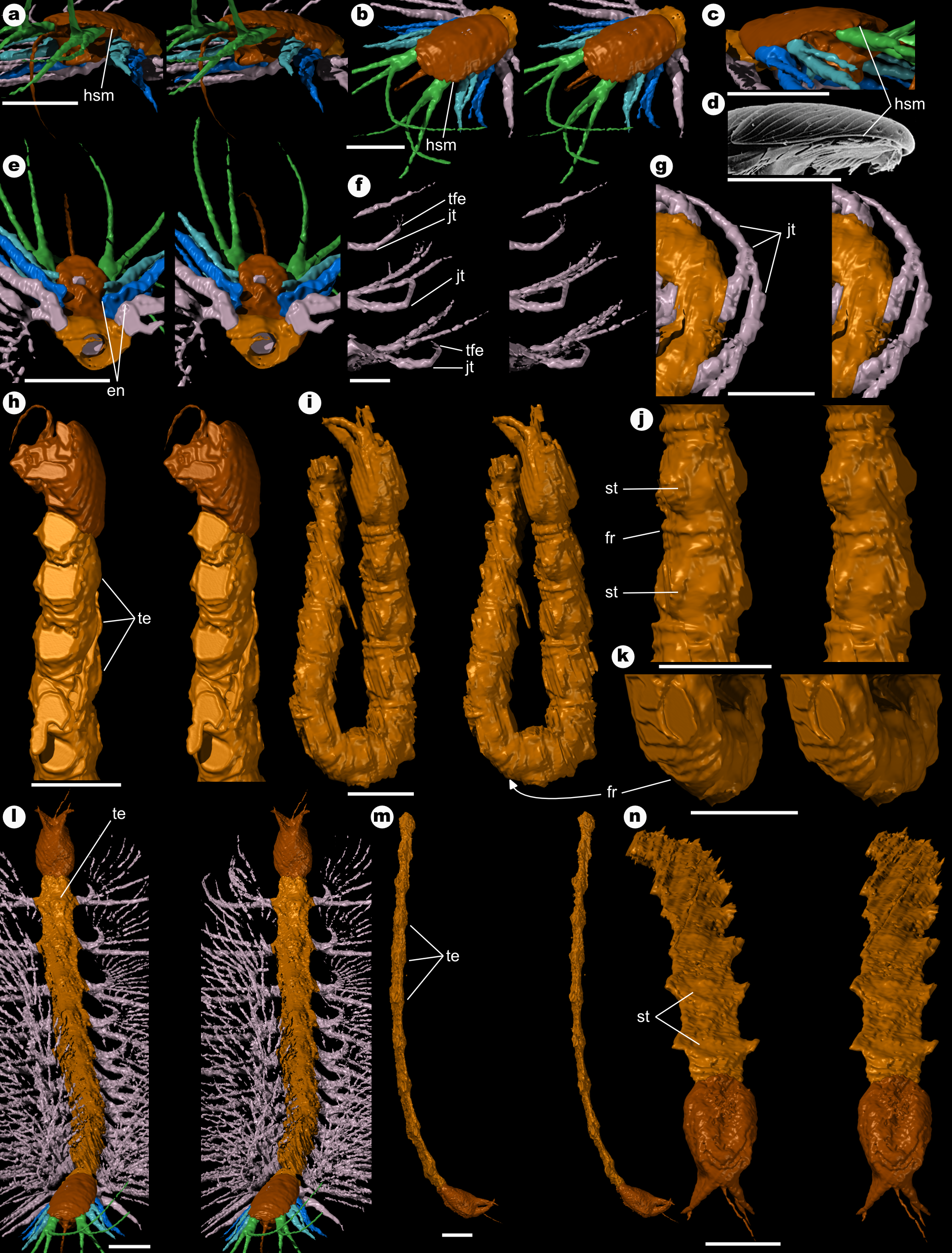

Supplement: ESM figure 1.png [file rspb20142663supp2.png]
